# Anencephaly and Severe Myelodysplasia in a Stillborn Brown Bear (*Ursus arctos arctos*)

**DOI:** 10.3390/ani12182345

**Published:** 2022-09-08

**Authors:** Ana Balseiro, Laura Polledo, José Tuñón, Juan Francisco García Marín

**Affiliations:** 1 Departamento de Sanidad Animal, Facultad de Veterinaria, Universidad de León, 24071 León, Spain; 2 Departamento de Sanidad Animal, Instituto de Ganadería de Montaña (CSIC-Universidad de León), Finca Marzanas, 24346 Grulleros, Spain; 3 Fundación Oso Asturias, 33114 Proaza, Spain

**Keywords:** brown bear, *Ursus arctos arctos*, congenital nervous system malformation, anencephaly, spina bifida, myelodysplasia, syringomyelia

## Abstract

**Simple Summary:**

A captive twenty-four-year-old female Eurasian brown bear (*Ursus arctos arctos*) gave birth to a stillborn cub at the end of gestation. Several malformations resulting from the anomalous development of the neural tube, not previously reported in bears, were observed in the cub. These included anencephaly, hypoplasia, micromyelia, severe myelodysplasia, syringomyelia, and spina bifida. The aetiology remains unidentified.

**Abstract:**

Malformations in the development of the neural tube have been described to be associated with different aetiologies, such as genetic factors, toxic plants, chemical products, viral agents, or hyperthermia. A twenty-four-year-old female Eurasian brown bear (*Ursus arctos arctos*), permanently in captivity and kept under food and management control, gave birth to a stillborn cub at the end of gestation. Several malformations resulting from the anomalous development of the neural tube, not previously reported in bears, were observed, such as anencephaly, hypoplasia, micromyelia, severe myelodysplasia, syringomyelia, and spina bifida. Multiple canal defects (e.g., absence) were also observed in the spinal cord. In some regions, the intradural nerve roots surrounded the spinal cord in a diffuse and continuous way. The aetiology remains unidentified, although the advanced age of the mother and/or folic acid deficit might have been the possible causes of this disorder. Supplements of folate given to the mother before and during early pregnancy may have reduced the incidence of neural tube defects. That supplementation should be considered when the reproduction of bears is to occur in captivity, in order to prevent the loss of future generations of this endangered species.

## 1. Introduction

Congenital malformations of the central nervous system (CNS) are frequently observed in mammals and are usually incompatible with life. The high degree of differentiation and the complexity of the CNS increase the susceptibility to developmental disorders. The congenital malformations of the CNS include failure of structural development, retardation of normal development, degeneration of formed tissues, or primary disturbances of function rather than of tissue structure [1]. The most frequent anatomic abnormalities are neural tube defects, which are initiated at the beginning of gestation in a narrow time range, usually from 8 to 28 days of gestation depending on the species [1].

The cause of malformations in individual cases is seldom determined, in part because of the long time lapse between the initiating event and the foetal presentation. In addition to inherited diseases, there are a large number of recognised environmental agents, both infectious and toxic, capable of causing anomalies [1,2].

The great majority of case records with anencephaly and other abnormalities of the CNS have been obtained from humans and cattle [1,2,3,4,5,6], although they have been described in several domestic animals, such as foals, lambs, or puppies [1,5]. However, knowledge of the congenital malformations of the CNS in free-ranging wildlife is limited [7] and remains unrecorded because of the difficulty in finding stillborn animals in nature. In addition, reports on captive wild animals are scarce and include disorders such as cerebral aplasia in one-horned rhinoceros (*Rhinoceros unicornis*) [8], hydrocephalus in baboons (*Papio* spp.) [9], or skull malformation and cerebellar herniation in African lions (*Panthera leo*) [10]. Here, we report a case of congenital malformation of the CNS in a Eurasian brown bear (*Ursus arctos arctos*) that was stillborn in captivity.

In Spain, the free-ranging Eurasian brown bear population is located in the Cantabrian Mountain range (northwestern Iberian Peninsula), representing the southwestern limit distribution for this species in Europe [11]. On an international level, the Cantabrian brown bear is listed on the IUCN Red List of Threatened Species and catalogued as in danger of extinction [12], with an estimated population of 320 individuals presently [13,14].

## 2. Materials and Methods

In the Principality of Asturias (northern Spain), a Brown Bear Rehabilitation Program is established when bears are found injured or alone (in the case of cubs) in nature. If rehabilitation is possible, animals are then reintroduced to the wild. However, when this is not possible, the animals remain in captivity. This was the case of our female brown bear, kept captive from 1989, when it was rescued as a cub (along with her sister) in Asturias. The bear was kept since then under food and management control until she died in 2018.

In 2013, the present female bear was twenty-four-year-old, and she gave birth to a stillborn cub at the end of gestation. The animal weighed 327 g. A complete postmortem examination of the carcass was conducted at the University of León (Spain), and gross lesions were recorded. Samples for histopathology were taken from the CNS (including sections of the spinal cord, one each from a different anatomical level—thoracic, lumbar, and sacral), as well as representative samples of the heart, lung, kidney, liver, spleen, gut, skeletal muscle, and vertebrae. Samples for histology were fixed in 10% neutral buffered formalin prior to trimming, and afterwards, they were routinely processed through graded alcohols and embedded in paraffin wax. Semi-serial 4 µm sections were cut, mounted on glass microscope slides, and stained with haematoxylin and eosin, Krüber-Barrera, and Masson’s trichrome stains. Prior to the histological routine, a decalcification solution (10% sodium citrate and 21% formic acid diluted in distilled water) was employed to remove mineral substances from vertebrae.

## 3. Results

**Figure 1:**
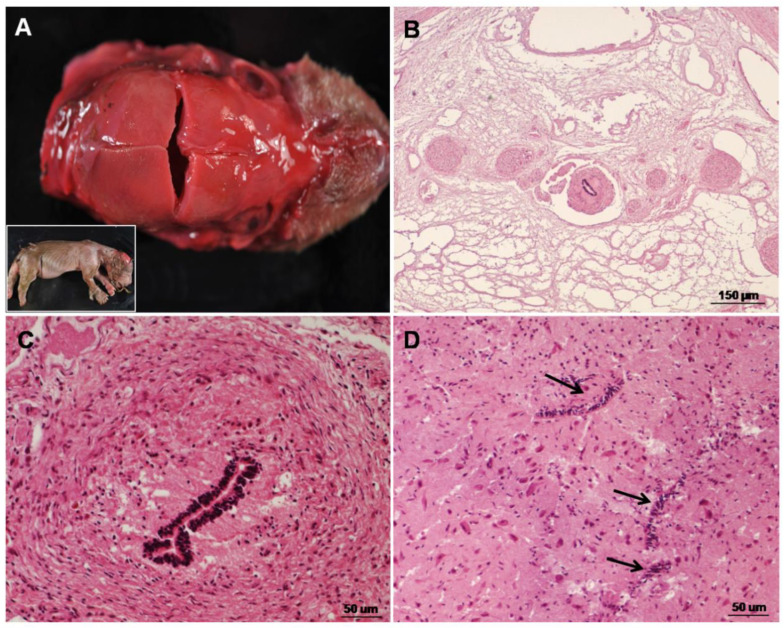
Gross (**A**) and microscopic (**B**–**D**) congenital malformations of the central nervous system in a stillborn Eurasian brown bear (*Ursus arctos arctos*): (**A**) stillborn female brown bear: Note cranium bifidum with opening of parietal and frontal bones of the skull, mainly at sutura coronalis level; (**B**) spinal cord: Severe hypoplasia and myelodysplasia are observed. Haematoxylin–eosin stain; (**C**) spinal cord: Cross-section showing a distorted canal partially lined by a pseudostratified layer of ependymal cells. Haematoxylin–eosin stain; (**D**) spinal cord: Presence of duplicated atresic central canals (arrows). Haematoxylin–eosin stain.

The macroscopical study showed cranium bifidum with an excessive opening of the parietal and frontal bones of the skull, mainly at the sutura coronalis level (Figure 1A). A complete anencephaly (absence of the brain) was also observed. Additionally, the absence of the cranial cervical spinal cord and some segments of the lumbar spinal cord could also be seen. Microscopically, the remaining spinal cord showed severe hypoplasia and myelodysplasia (abnormal development of the spinal cord) characterised by the loss of normal structure (Figure 1B). Several central canal defects such as the absence, atresia, or presence of multiple canals were present in the spinal cord (Figure 1C,D). In some sections, a distorted canal partially lined by a pseudostratified layer of ependymal cells was observed (Figure 1C). Diffuse micromyelia, lack of ventral median fissure, severely diminished diameter with a reduction in grey and white matter, and lack of nervous nuclei or low neuron presence were also observed in the spinal cord.

**Figure 2:**
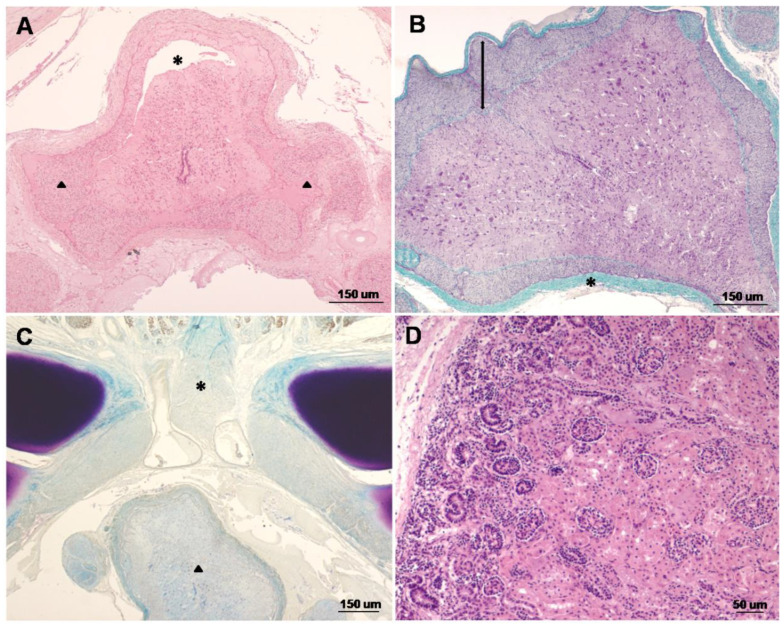
Histopathological findings in a stillborn Eurasian brown bear (*Ursus arctos arctos*) showing congenital malformations of the central nervous system: (**A**) lumbar spinal cord: Presence of meningomyelocele (arrowheads), syringomyelia (asterisk), and an atresic central canal. Note the lack of differentiation of grey and white matter, the absence of nervous nuclei, and the presence of few neurons. Haematoxylin–eosin stain; (**B**) lumbar spinal cord: Dura mater shows fibrosis (asterisk), and intradural nerve roots surround the spinal cord in a diffuse and continuous way (arrow). Masson’s trichrome stain; (**C**) caudal lumbar segment of the spinal cord: Note spina bifida (asterisk) with lack of closure of dorsal bones and presence of demyelination without inflammation in spinal cord (arrowhead). Absence of central canal and failure of ventral medial fissure in the spinal cord are also observed. Krüber–Barrera stain; (**D**) kidney: Moderate renal hypoplasia can be observed. Haematoxylin–eosin stain.

Syringomyelia (tubular cavitation of the spinal cord) was present in different sections of the lumbar spinal cord always located in dorsal funiculi (Figure 2A). Meninges were present in all areas of the CNS, accompanied by the intradural and extradural nerve roots. The dura mater showed fibrosis throughout the spinal cord. In some regions, the intradural nerve roots surrounded the spinal cord in a diffuse and continuous way (Figure 2B). Spina bifida (absence of the dorsal portions of the vertebrae) and meningomyelocele (meninges and spinal cord protrude) were observed in the caudal lumbar segment of the spinal cord (Figure 2C). Demyelination without inflammation was seen along the length of the spinal cord by using Krüber–Barrera staining (Figure 2C). Congenital lung atelectasis and moderate renal hypoplasia were also present (Figure 2D). No other gross or microscopic abnormalities were observed.

## 4. Discussion

The lesions reported here have not been previously described in bears and include several congenital malformations of the CNS related to the defective development of the neural tube. Most of those lesions have been previously described in other mammals to be associated with different causes or with an unexplained origin [1,2,3,4,5,6], but they have been scarcely reported in wildlife (captive or free-ranging). In this case, the complete absence of the brain (“true anencephaly”) is remarkable, as it is very rarely found [2,3,4,5]. The closure of the neural tube is an essential step in the development of the CNS [15,16]. Failure of closure in the early stages of brain formation and an abnormality of mesenchymal structures result in neural tube defects, including anencephaly, cranium bifidum, and spina bifida. Some neural tube defects affecting the brain (e.g., anencephaly) are invariably lethal perinatally [3]. Others, such as myelodysplasia, can be lethal or produce severe clinical signs [6], whereas open spina bifida is compatible with postnatal survival but frequently results in serious handicap [1,2].

Investigation into the aetiology of neural defects is complicated by the fact that the same type of abnormality may be produced by both genetic or chromosomal and exogenous causes. There are different exogenous causes of CNS malformations such as exposure to infectious agents and other teratogens [1], certain therapeutic agents, toxic plants [6], vitamin A administered during gestation [17], and hyperthermia [18,19]. The mutation of more than 200 genes is also known to cause neural tube defects in mice [20].

The outcome of foetal interactions with agents potentially teratogenic for the nervous system depends on the species and age of the foetus, but also on the nature of the agent and its cellular tropism, commonly directed toward immature rapidly dividing cells [1]. The most common mode of action for teratogens is the selective destruction of cells. These cytolytic effects have been demonstrated with neural defects induced by viruses (e.g., orthobunyaviruses, orbiviruses, or pestiviruses)—as a direct consequence of infection or developed as part of the inflammatory reaction—and chemicals, as well as by physical agents such as hyperthermia [1,17,18,19].

The aetiology in this case remains unidentified. The mother was always kept in captivity under control and neither therapeutic nor pharmaceutical treatment nor teratogenic plants nor toxics were administrated or taken during gestation, and she was always in a healthy state. Moreover, no lesions associated with some of the former aetiologies (i.e., viral infection) were observed in the bear. The advanced age of the mother can increase the risk of these congenital anomalies [2]. It might be hypothesised that the very advanced maternal age (brown bear lifespan is 22 to 32 years in the wild or captivity) could have been the cause of the disorder, although we cannot confirm it, because other aetiologies such as viral infections or toxics were not discarded using specific diagnostic techniques.

On the other hand, the prevention of neural tube defects became a realistic proposition, following the finding that maternal supplementation with folic acid is an effective method for the primary prevention of a proportion of neural tube defects, in both humans and mice, although the embryonic mechanism of folate action remains unclear [20,21,22]. Therefore, supplements of folate given to the mother before and during early pregnancy may have reduced the incidence of neural tube defects. That supplementation should be taken into account when the reproduction of bears is to occur in captivity, in order to prevent the loss of future generations of this endangered species.

## 5. Conclusions

Anencephaly and severe myelodysplasia were described in Eurasian brown bear, lesions that should be considered when the reproduction of this endangered species is carried out in captivity.

## Data Availability

Not applicable.

## References

[1] Maxie M.G. (2016). Malformations of the central nervous system. Jubb, Kennedy and Palmer’s Pathology of Domestic Animals.

[2] Summers B.A., Cummings J.F., de Lahunta A. (1995). Malformations of the central nervous system. Veterinary Neuropathology.

[3] Cho D.Y., Leipold H.W. (1978). Anencephaly in calves. Cornell Vet..

[4] Leipold H.W., Hiraga T., Dennis S.M. (1993). Congenital defects of the bovine central nervous system. Vet. Clin. N. Am. Food Anim. Pract..

[5] Washburn K.E., Streeter R.N. (2004). Congenital defects of the ruminant nervous system. Vet. Clin. N. Am. Food Anim. Pract..

[6] Polledo L., García Marín J.F., Martínez-Fernández B., González J., Alonso J., Salceda W., García-Iglesias M.J. (2012). Recurrent outbreaks of myelodysplasia in newborn calves. J. Comp. Path..

[7] Rawlins R.G., Kessler M.J. (1983). Congenital and hereditary anomalies in the rhesus monkeys (*Macaca mulatta*) of Cayo Santiago. Teratology.

[8] Schaftenaar W., Fernandes T., Fritsch G., Frey R., Szentiks C.A., Wegner R.D., Hildebrandt T.B., Hermes R. (2011). Dystocia and fetotomy associated with cerebral aplasia in a greater one-horned rhinoceros (*Rhinoceros unicornis*). Reprod. Domest. Anim..

[9] Fox B., Owston M.A., Kumar S., Dick E.J. (2011). Congenital anomalies in the baboon (*Papio* spp.). J. Med. Primatol..

[10] Baker J.R., Lyon D.G. (1977). Skull malformation and cerebellar herniation in captive African lions. Vet. Rec..

[11] Naves J., Wiegand T., Fernández A., Stephan T. (1999). Riesgo de Extinción del Oso Pardo Cantábrico.

[12] McLellan B.N., Proctor M.F., Huber D., Michel S., IUCN SSC Bear Specialist Group Brown bear (Ursus arctos) Isolated Populations (Supplementary Material to Ursus arctos arctos Redlisting Account). The IUCN Red List of Threatened Species 2017, e.T41688A121229971. https://www.iucnredlist.org/species/41688/121229971.

[13] González E.G., Blanco J.C., Ballesteros F., Alcaraz L., Palomero G., Doadrio I. (2016). Genetic and demographic recovery of an isolated population of brown bear *Ursus arctos* L., 1758. PeerJ.

[14] Fundación Oso Pardo. https://fundacionosopardo.org/.

[15] Padmanabhan R. (1984). Experimental induction of cranischisis aperta and exencephaly after neural tube closure: A rat model. J. Neuropathol. Exp. Neurol..

[16] Dewey C.W., Marino D.J., Loughin C.A. (2013). Craniocervical junction abnormalities in dogs. N. Z. Vet. J..

[17] Prozesky L., Joubert J.P.J., Ekron M.D. (1981). Paralysis in lambs caused by overdosing with parbendazole. Onderstepoort J. Vet. Res..

[18] Edwards M.J. (1983). Hyperthermia and birth defects. Cornell Vet..

[19] Graham J.M. (2020). Update on the gestational effects of maternal hyperthermia. Birth Defects Res..

[20] Copp A.J., Greene N.D. (2010). Genetics and development of neural tube defects. J. Pathol..

[21] Smithells R.W., Sheppard S., Schorah C.J., Seller M.J., Nevin N.C., Harris R., Read A.P., Fielding D.W. (1980). Possible prevention of neural-tube defects by periconceptional vitamin suplemmentation. Lancet.

[22] Seelan R.S., Mukhopadhyay P., Philipose J., Greene R.M., Pisano M.M. (2021). Gestational folate deficiency alters embryonic gene expression and cell function. Differentiation.

